# The Correlation Between Emotionality Changes and Alcohol Consumption in Young Persons: A Pilot Study

**DOI:** 10.3390/healthcare13090987

**Published:** 2025-04-24

**Authors:** Simona Dana Mitincu-Caramfil, Lavinia-Alexandra Moroianu, Andrei Vlad Bradeanu, Oana-Maria Isailă, Cecilia Curis, Eduard Drima

**Affiliations:** 1 Department of Pharmaceutical Sciences, Dunărea de Jos University, 800201 Galați, Romania; simona_caramfil@yahoo.com (S.D.M.-C.); lavinia.moroianu@yahoo.com (L.-A.M.); 2 Surgical Department, Dunărea de Jos University, 800201 Galați, Romania; andrei.bradeanu@ugal.ro; 3 Department of Legal Medicine and Bioethics, Faculty of Dental Medicine, “Carol Davila University” of Medicine and Pharmacy, 020021 Bucharest, Romania; 4 Medical Department, Dunărea de Jos University, 800201 Galați, Romania; 5 Clinical Medical Department, Dunărea de Jos University, 800201 Galați, Romania; drima_edi1963@yahoo.com

**Keywords:** alcohol, young persons, mental disorders

## Abstract

**Background/Objectives:** Alcohol consumption in young persons is a growing phenomenon, with significant implications for physical and mental health. This behavior exposes adolescents and young adults to multiple risks, such as affecting cognitive functions, the development of emotional disorders, and social integration difficulties. The present study sets out to investigate the way alcohol consumption influences emotionality in young people, focusing on the emotional changes (anxiety and irritability), cognitive changes (attention deficit and memory disorder), and behavioral changes (impulsivity and aggressiveness). **Methods:** The methodology involved collecting quantitative data from a sample of young people who consume and do not consume alcohol, using standardized questionnaires and advanced statistical software (processed in MATLAB version 9.11_R2021b). We analyzed variables such as consumption frequency and intensity, affective scores, and demographic factors to highlight the correlations between consumption level and the intensity of affective modifications. **Results:** The results revealed a significant association between alcohol consumption and the rise in anxiety and depression symptoms or a tendency toward impulsive behaviors. Additionally, we observed that psychosocial factors, including group pressure, family climate, and stressful contexts, can exacerbate emotional vulnerability. **Conclusions:** This study suggests the need for early psychological interventions and prevention programs to approach emotional and cognitive dimensions and the social influences associated with consumption. Implementing support and counseling strategies, as well as education and awareness campaigns, can contribute to reducing risky behavior and promoting young people’s harmonious development.

## 1. Introduction

Alcohol consumption in young people is a major problem worldwide, with complex consequences on their physical and mental health [1,2,3]. In Romania, the amplitude of this phenomenon is alarming. According to the 2012 national report by the Romanian Anti-Drug Agency (Agenția Națională Antidrog), experimental alcohol use—often in combination with prescription substances—has a high prevalence among 16-year-old students and is reported across all regions of the country [3,4].

The effects of excessive alcohol consumption on the physical health of young people are numerous and potentially severe, including liver, cardiovascular, and gastrointestinal disorders [5]. Furthermore, alcohol use during adolescence may negatively impact brain development, with long-term repercussions on cognitive functions such as memory, attention, and decision-making, as well as behavioral regulation.

From a psychiatric perspective, alcohol consumption during adolescence is associated with a high incidence of mental disorders, such as depression and anxiety [5]. Moreover, there is proof that exposing youngsters to psychoactive substances can increase the risk of psychotic disorders, including schizophrenia, and it can amplify the risk of suicide, significantly affecting cognitive and social functionality [6].

Although the specialized literature includes multiple studies on alcohol consumption in adolescents, there are important gaps regarding the prevalence and characteristics of mental disorders among young people in Romania [3]. In particular, existing data are often old or are generated from self-evaluation assessments that are less sensitive than structured clinical interviews. This lack of updated information underlines the need for rigorous research meant to underlie the developing strategies of prevention and intervention adapted to the young population’s specific character [3].

Filling these gaps through relevant epidemiological and clinical investigations represents a priority for sketching an efficient public policy and for implementing health programs meant to reduce alcohol consumption and support young people’s mental health.

### 1.1. Psychosocial Context

#### 1.1.1. Social, Environmental, and Cultural Factors

Alcohol consumption in young people is determined by a series of social and environmental factors, of which the pressure exerted by the social group takes the main place [7]. In trying to obtain integration and validation from their colleagues, adolescents can adopt harmful consumption habits, influenced by the attitudes and behaviors of those around them. Positive expectations regarding alcohol consumption, risk-oriented personality traits, and cultural norms can also contribute to an increased vulnerability toward alcohol [8]. In certain cultural contexts, the community’s habits and values can encourage or tolerate higher consumption; for example, in young Americans of Asian origins, the psychosocial, cultural, and genetic factors proved to have a strong influence over consumption behavior [9].

#### 1.1.2. Family Factors

The family environment and parental style play an essential role in initiating and maintaining alcohol consumption in adolescents [10]. The behavioral models to which young people are exposed, as well as parents’ attitudes toward alcohol, can determine the children’s attitudes toward substance consumption [11]. In addition, the presence of antisocial friends and delinquent behaviors was identified as a major predictor of substance consumption in adolescents, underlining the importance of family and social mechanisms in prevention [12].

#### 1.1.3. Psychological Perspectives on Personality Changes and Emotionality

At a psychological level, alcohol consumption in young people can generate personality and emotional changes, reflected in the increase in impulsivity, emotional instability, and diminished self-control capacity [13]. Also, studies that used the Five-Factor Model (FFM—Five-Factor Model [14]) suggest that diminished conscientiousness and emotional stability in drunkenness periods are correlated to an enhancement of negative consequences associated with alcohol consumption [13]. These youngsters often have a reduced tolerance to stress and adopt inefficient coping strategies that can fuel a vicious consumption and emotional vulnerability cycle.

Understanding the psychosocial context—including social, cultural, and family influences—is essential in developing efficient prevention and intervention programs that both lower external pressures and improve youngsters’ psychological resources.

#### 1.1.4. Emotional Dysregulation and Affective Instability

Emotional dysregulation refers to difficulties in managing emotional responses appropriately and flexibly, often resulting in mood fluctuations, irritability, impulsivity, and aggression. These emotional and behavioral changes can impair interpersonal relationships and adaptive functioning, especially during adolescence. In the context of alcohol consumption, young individuals may experience increased emotional reactivity and a diminished capacity for emotional self-regulation. Alcohol, acting as a central nervous system depressant, can initially blunt emotional discomfort but subsequently worsen affective instability and impulsivity, thus creating a vicious cycle of self-medication and worsening emotions. Studies indicate that adolescents with poor emotional regulation are more prone to initiate and maintain alcohol use, often in response to anxiety, stress, or depressive moods [15,16].

Alcohol consumption during adolescence and early adulthood is tightly connected to emotional modifications. Alcohol acts as a depressant of the central nervous system, negatively influencing cognitive and emotional functions [17]. Adolescents and young people can resort to alcohol consumption to deal with depression, anxiety, shyness, or stress [16]. However, using alcohol as an emotional adjustment mechanism can lead to addiction and aggravate existing emotional problems [18]. Studies show that persons who start consuming alcohol before the age of 15 present a 4 times higher risk of developing alcohol addiction compared to those who start consuming alcohol after the age of 20 [16]. Moreover, precocious alcohol consumption can affect brain development, with long-term consequences on emotional and behavioral control [16].

Understanding the concept of emotional alteration and its connection with alcohol consumption in young people is important for developing efficient prevention and intervention strategies. The therapeutic approach should focus on identifying and managing emotional factors that lead to alcohol consumption, promoting healthy mechanisms of emotional adjustment at the same time [18].

## 2. Materials and Methods

The present methodology was conceived to investigate the emotionality alterations in young alcohol consumers in the context of psychosocial and clinical factors. We pursued both descriptive aspects (prevalence, demographic characteristics, and types of consumption) and correlational aspects, aiming for the relation between alcohol consumption level and emotional indicators (anxiety, depression, and irritability). The statistical analysis was carried out in MATLAB version 9.11_R2021b to quantify and interpret possible significant associations. 

The main objectives pursued in this research study were as follows:

- Investigating the relationship between the level of alcohol consumption and emotional indicators such as anxiety and depression;
- Analyzing differences between youngsters with low, moderate, and high alcohol consumption concerning their emotional state and coping mechanisms;
- Exploring, based on the existing literature, the influence of family and social context on the emotional vulnerability associated with alcohol use.

Hypotheses of the study

1. Young people who report high alcohol consumption score significantly higher on the anxiety and depression scales compared to occasional or non-consumers.
2. A higher frequency of alcohol consumption episodes is associated with increased levels of depressive symptoms.
3. Family-related factors (e.g., permissive parental attitudes or parental consumption models) may amplify emotional symptoms according to previous findings.

### 2.1. Design of the Research

The present study was designed as a cross-sectional, observational, and correlational investigation aimed at identifying and analyzing the relationship between alcohol consumption and emotional changes in young individuals. The subjects were selected in the time frame of January 2021–December 2023, taking into account the availability of relevant clinical and socio-demographic data, as well as informed consent.

Through its transverse nature, this study reveals the situation relative to a precise moment, allowing the identification of correlations between the frequency/quantity of alcohol consumption and emotionality indicators (depression, anxiety, mood, irritability, etc.). The observational design does not imply interventions or experimental manipulations but follows the participants’ natural evolution and the association of the studied factors, and the correlational component facilitates the examination of the statistical connection between the variables.

The data were collected during hospitalization/consultations over six months, a time interval long enough to comprise a heterogeneous sample of young people presenting the distinct stages and types of alcohol consumption. This approach allows us to see subjective aspects (such as the reported emotional state) and objective ones (clinical parameters) at the same point in time. Also, the geographic location (Galati County) and the hospital environment not only offer an adequate frame for recruiting participants but also a specific perspective on the mental health problems associated with alcohol consumption in young people.

### 2.2. Participants

The sample included youngsters aged between 18 and 30 who were hospitalized or on record uat the “*Elisabeta Doamna*” Psychiatric Hospital in Galati. The inclusion criteria were as follows:

- The ability to give informed consent;
- The presence of clinical and socio-demographic data belonging to the 18–30 age category or closer.

The exclusion criteria included the presence of severe neurocognitive or psychiatric conditions (e.g., psychotic disorders, dementia, or intellectual disability) that could compromise the accuracy of emotional self-reporting.

The selection of the participants was carried out through a convenience non-probabilistic method given the specificity of the psychiatric institution and the accessibility of the people in the target group. Thus, the study sets out to describe and understand the relationship between alcohol consumption and the emotional changes in this cohort without strictly generalizing to the entire population of young consumers.

### 2.3. Instruments and Variables

The collected data contain information on the socio-demographic and psychological variables collected from the included youngsters aged between 18 and 30. From the data, we learn aspects concerning their general clinical state, as well as the scores obtained by applying specific instruments of psychological evaluation. Of the included variables, we have the participants’ sex, the provenance environment, the level of studies, and also their state at discharge from the hospital, reflecting the patient’s evolution at the end of the monitoring period (for example, improved state, partially improved, etc.).

Other relevant variables target self-esteem, described by self-reported or observed levels; the stability of character traits, and scores obtained using standardized psychological instruments. The Beck Depression Inventory (BDI) [19] offers a numeric indicator of depressive symptoms, and the State-Trait Anxiety Inventory (STAI) [20] measures state anxiety (S) and trait anxiety (STAI_T), where higher scores indicate accentuated anxiety. Additionally, we included the variables AffectPoz (positive affect) and AffectNeg (negative affect), which were derived from structured psychological observations during hospitalization. These variables reflect the clinician-reported emotional predisposition of participants but do not originate from a standardized or validated instrument. As such, they were treated as exploratory indicators, and their interpretation was carried out with caution.

Alcohol consumption was categorized based on self-reported frequencies:

- *Occasional*: less than once per week;
- *Moderate*: 1–3 times per week;
- *Heavy*: more than 3 times per week or binge drinking episodes (defined as 5+ drinks on a single occasion).

These thresholds were adapted from existing epidemiological studies on youth drinking patterns [8].

The emotionality analysis in this context was carried out by evaluating depression (BDI), anxiety (STAI), and positive/negative emotionality, with these dimensions being considered relevant in understanding the impact of alcohol consumption on young people’s psychological and emotional state. The instruments used, such as the Beck Depression Inventory and State-Trait Anxiety Inventory, are standardized methods frequently applied in psychological research, facilitating an objective quantification of depressive and anxious symptoms.

### 2.4. Procedure

The data were collected through a mixed approach, combining information from the patient’s medical files with direct psychological evaluations. In the first stage, doctors and psychologists from the “Elisabeta Doamna” Psychiatric Hospital centralized demographic and clinical data from the patients’ files (age, provenance environment, level of studies, and history of alcohol consumption), filling in the variables about the mental health state and the evolution at discharge.

Simultaneously, when the clinical state allowed it, the patients were invited to participate in short face-to-face interviews, during which standardized psychological instruments (BDI and STAI) were applied. The answers were directly registered in the observation charts and later introduced in the working file. For persons with difficulties in expressing themselves or those reticent in offering detailed information, we turned to additional data from existing clinical files, thus avoiding the duplication of information.

From an ethical point of view, the study respected confidentiality norms and the protection of personal data, following the legislation in effect and the hospital’s internal regulations. The participants and/or their legal guardians were informed about the purpose of this research study, the collection and data processing procedure, and their right to withdraw at any time, without any consequence on their therapeutic plan. For all cases, we obtained informed consent. This study was approved by the Ethics Committee of Elisabeta Doamna Psychiatry Hospital (protocol code PO 22-STUD01/10, No 4, date of approval: 22 October 2019)

The psychological instruments were administered by licensed clinical psychologists with training in standardized assessment procedures. Evaluations were conducted within the psychiatric hospital setting, primarily during inpatient care. Assessments were performed only when the patients’ mental state was clinically evaluated as stable enough to allow accurate self-reporting and participation. These measures ensured the reliability of data collection and minimized the influence of acute symptomatology.

### 2.5. Statistical Analysis

To process the data and execute statistical significance tests, we used the MATLAB software version 9.11_R2021, with available statistical analysis packages. In the first stage of this analysis, we calculated the descriptive indicators, such as the mean, standard deviation, frequencies, and percentages, for the key variables, using BDI, STAI_S, STAI_T, AffectPoz, and AffectNeg, respectively. Later, we applied specific tests to verify the research hypothesis.

The relation between the alcohol consumption level, classified as occasional, moderate, and abusive, and the scores obtained from the psychological scales of depression, anxiety, and emotionality was explored through the Pearson or Spearman correlation tests. Comparisons of the medium of the groups were carried out via ***t*-tests** for independent samples or Variation Analysis (ANOVA). This was carried out in situations in which differences needed to be highlighted as far as BDI scores were concerned: for example, between consumers with low self-esteem and those with average self-esteem or between subjects in rural environments and urban environments.

In the situations where the data did not fulfill normality conditions, we used non-parametric tests, such as Mann–Whitney U and Kruskal–Wallis. For a more profound understanding of the multiple interactions between the variables, we applied models of linear or logistic regression, for example, to estimate the simultaneous impact of self-esteem and the condition at discharge on the BDI score.

The threshold of significance was established at *p* < 0.05, and the effects under this value were considered significant from a statistical point of view. In analyzing the results, we also considered the trust intervals, as well as the effect’s size, using indicators such as Cohen’s d and ŋ^2^ where relevant.

## 3. Results

The study included a total of 60 participants, of whom 41 were male (68.3%) and 19 were female (31.7%). Regarding their education level, 22 participants (36.7%) had completed only primary education, while 38 participants (63.3%) had received secondary education, reflecting the socio-educational diversity within the sample.

This demographic and educational structure provides a relevant framework for investigating the impact of alcohol consumption on emotionality among young individuals and for exploring the potential mediating or moderating factors that influence this relationship.

### 3.1. Alcohol Consumption Distribution

**Figure 1:**
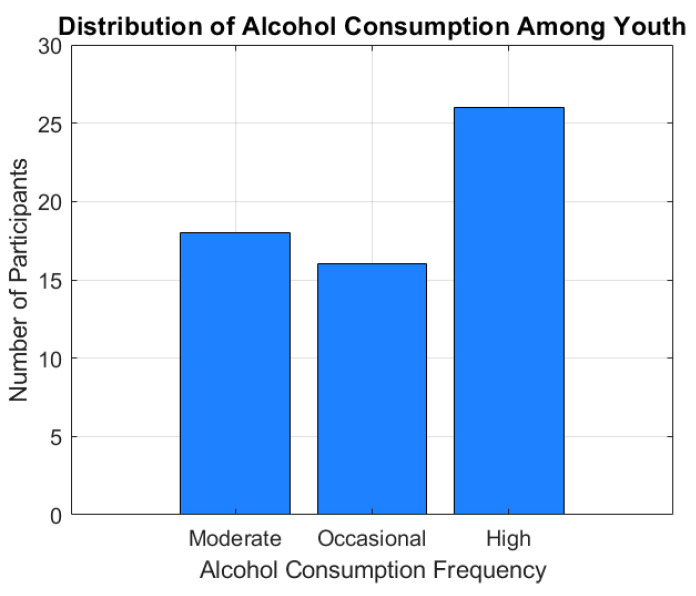
Distribution of alcohol consumption among youth.

The alcohol consumption distribution is represented using a category histogram (Figure 1). This shows the distribution of participants according to consumption categories (occasional, medium, and high).

Figure 1 illustrates the distribution of alcohol consumption frequencies among the participants, categorized as moderate, occasional, and heavy consumers. Out of the total sample, 18 individuals (30%) reported a moderate level of alcohol consumption, 16 participants (26.7%) consumed alcohol occasionally, while 26 subjects (43.3%) fell into the heavy consumption category.

The distribution reveals a marked predominance of heavy drinkers within the studied group, which may reflect patterns of risky behavior, reduced supervision, or the increased availability of alcohol among youth. These trends appear particularly accentuated in participants aged over 20, suggesting a link between age, autonomy, and alcohol use intensity.

### 3.2. Age Distribution of Participants

**Figure 2:**
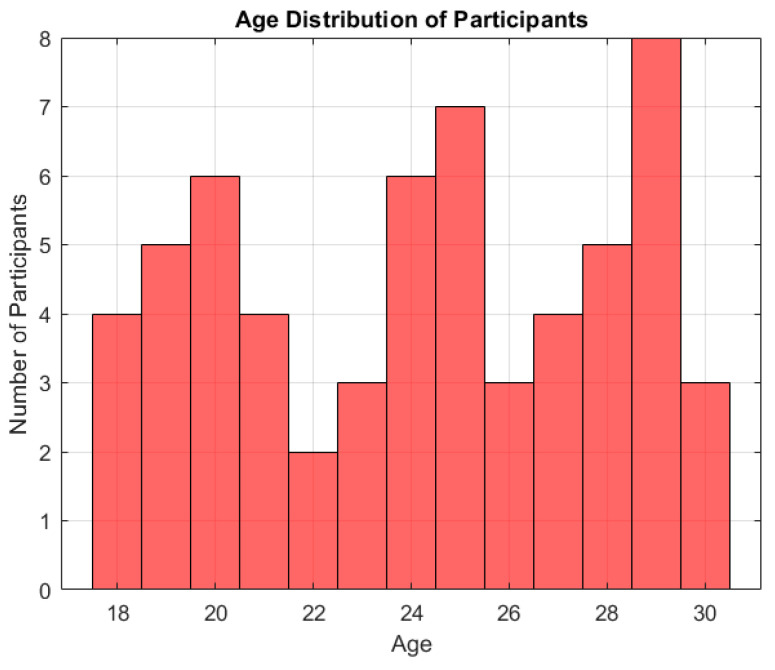
Age distribution of participants in the analyzed sample.

The participants’ average age was M = 24.18 years (SD = 3.80). The age distribution is visualized in Figure 2. Most participants were concentrated in the 20–24 age range, reflecting the study’s focus on young adults. Specifically, the breakdown by age intervals was as follows:

- 18–20 years: 12 participants (20%);
- 21–22 years: 16 participants (26.7%);
- 23–24 years: 14 participants (23.3%);
- 25–26 years: 9 participants (15%);
- 27 years and above: 9 participants (15%).

This distribution suggests that over two-thirds of the sample (70%) were aged between 18 and 24, a critical developmental period marked by increased emotional reactivity and vulnerability to risk behaviors such as excessive alcohol consumption.

Figure 2 illustrates the age distribution of the participants. Specifically, 12 individuals (20%) were aged 18–20, 16 (26.7%) were 21–22 years old, 14 (23.3%) were 23–24 years old, 9 (15%) were aged 25–26, and 9 (15%) were aged 27 or older. These results indicate that the majority of the sample (70%) falls within the 18–24 age range, a critical period in emotional and behavioral development.

### 3.3. Emotional Symptoms Appearance Rate

To evaluate the impact of alcohol consumption on emotionality, we analyzed the following psychological variables:

- BDI (Beck Depression Inventory) for depression;
- STAI_S (State Anxiety Inventory) for state anxiety;
- STAI_T (Trait Anxiety Inventory) for trait anxiety;

The descriptive statistics for these variables are presented in Table 1.

### 3.4. The Correlation Between Alcohol Consumption and Emotional Symptoms

**Figure 3:**
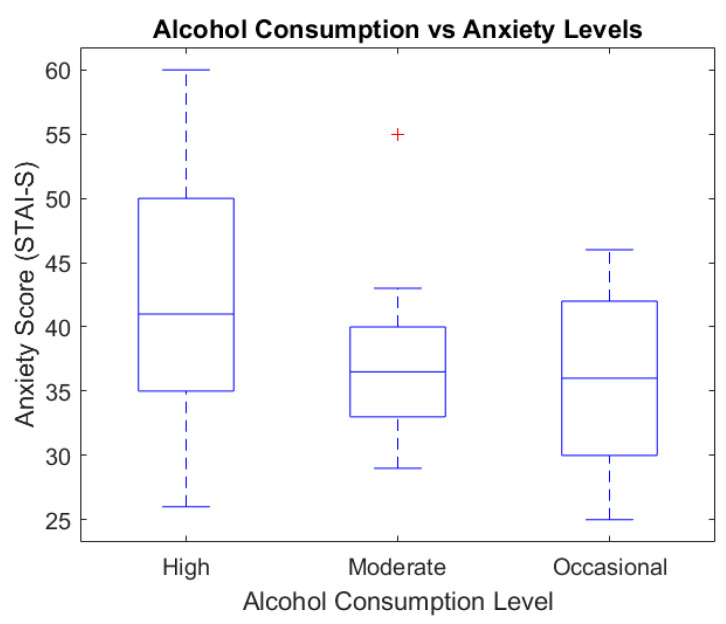
Alcohol consumption vs. anxiety levels (STAI_S).

**Figure 4:**
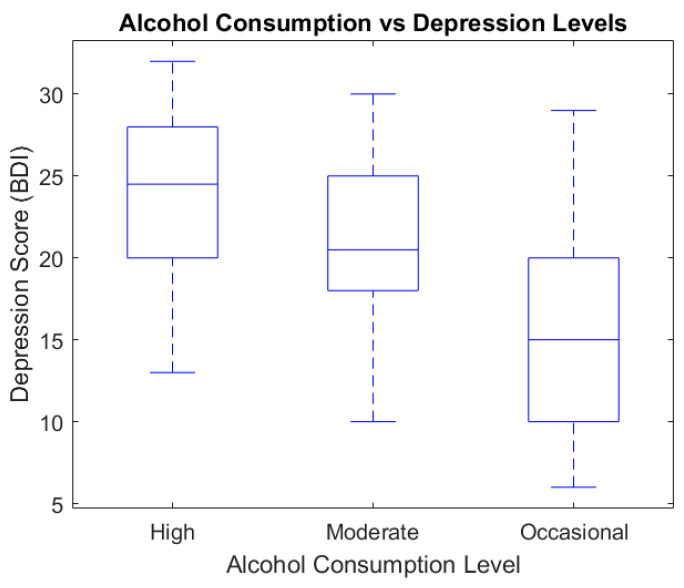
Alcohol consumption vs. depression levels (BDI).

To analyze the relationship between alcohol consumption and emotional symptoms, we used box plots and scatter plot graphic representations (Figure 3 and Figure 4).

Figure 3 presents the relationship between alcohol consumption and anxiety levels, as assessed via the STAI-S questionnaire. The highest median anxiety score was recorded in the heavy consumption group (median ≈ 43), followed by the moderate group (median ≈ 37), while the occasional group showed the lowest levels (median ≈ 34). These findings suggest a positive association between the intensity of alcohol use and self-reported anxiety symptoms.

The boxplot diagram below illustrates the STAI_S score distribution depending on the alcohol consumption level.

Figure 4 shows the variation in depressive symptom severity according to alcohol consumption levels, as measured via the BDI. Participants with heavy alcohol use reported the highest median depression score (median ≈ 25) compared to the moderate group (median ≈ 21) and the occasional group (median ≈ 14). This trend may reflect the potential aggravating role of alcohol on depressive symptomatology, reinforcing the importance of integrated therapeutic interventions.

The scatter plot diagram indicates the relationship between alcohol consumption and depression.

**Figure 5:**
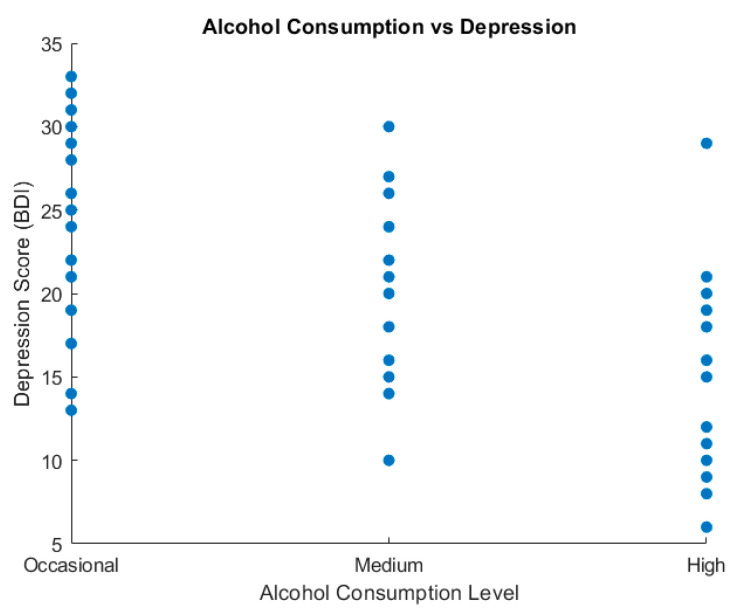
Alcohol consumption vs. depression (scatter plot).

Figure 5’s scatter plot illustrates the distribution of depression scores (BDI) across various levels of alcohol consumption (occasional, moderate, and high). Each dot represents an individual participant. Higher depression scores appear more frequently among individuals reporting high and moderate alcohol use, whereas occasional consumers tend to report lower BDI scores. This visual dispersion supports the observed association between increased alcohol intake and elevated depressive symptoms.

### 3.5. Inferential Analysis

To identify the associations between alcohol consumption and emotional changes evaluated with the BDI scale (depression) with respect to STAI (STAI_S and STAI_T), we appealed to Pearson correlations, ANOVA unifactorial tests, and linear regression models. The data come from the sample analyzed in MATLAB version 9.11_R2021.

The Pearson correlations between alcohol consumption and the scores relative to emotionality scales (BDI < STAI_S and STAI_T) were statistically significant and negative:

- Depression (BDI): r = −0.57; *p* < 0.001.
- State anxiety (STAI_S): r = −0.38; *p* = 0.003.
- Trait anxiety (STAI_T): r = −0.42; *p* = 0.001.

These values indicate that, in the studied sample, the higher scores for alcohol consumption are associated with lower reported levels of depression and anxiety, suggesting either a possible subjective “self-treatment” effect or associated confusion factors (for example, socio-demographic variables or denial mechanisms), an aspect that requires prudent interpretation.

To examine the differences between groups (non-consumers vs. moderate consumers vs. heavy consumers), we used unifactorial ANOVA:

- BDI: *p* < 0.001.
- STAI_S: *p* = 0.010.
- STAI_T: *p* = 0.003.

**Figure 6:**
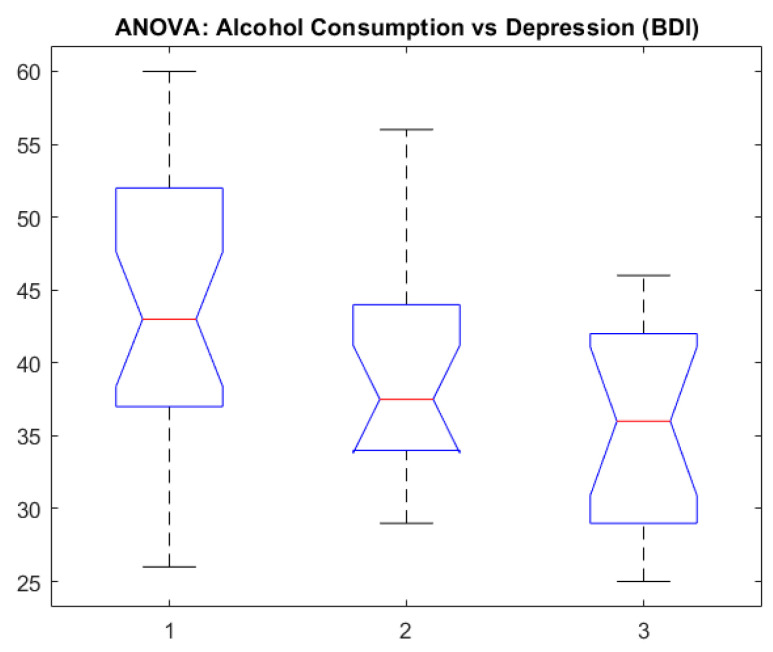
ANOVA: alcohol consumption vs. depression.

The results show significant differences between groups, confirming the assumption that there are variations in emotionality given the alcohol consumption level. With depression (BDI), the post hoc Tukey test showed a significant contrast between non-consumers and heavy consumers (*p* < 0.05). In Figure 6 (“ANOVA: AlcoholConsumptionDepression (BDI)”), we can observe the average differences in the depression scores for each level of alcohol consumption.

In order to explain the variations in the BDI, STAI_S, and STAI_T scores based on alcohol consumption (and on supplemental factors like sex and provenance environment), we built three regression models. With depression (BDI), the model presented a determination coefficient of R^2^ = 0.351 (*p* < 0.001), showing that approximately 35% of the BDI score variation is explained by the included variables. The coefficient associated with alcohol consumption was negative and significant (β = −5.17; *p* < 0.001), suggesting a smaller BDI score in individuals who declare greater alcohol consumption, after the sex and the residence environment data.

Similar models for anxiety (STAI_S and STAI_T) also indicated a negative relation between consumption and anxiety scores, although with a reduced explanatory power (R^2^ between 0.166 and 0.186). In both models, *p* was under 0.01. Although the statistics are significant, the clinical interpretation must be approached with caution, because there is a possibility of underreporting emotional symptoms in participants with chronic consumption or other contextual factors that were not included in the model.

In conclusion, the inferential analysis backs the idea that there are significant differences in the emotionality parameters between various levels of alcohol consumption, and this underlines the need for supplemental investigations, possibly longitudinal investigations, in order to establish the causal sense and clinical significance of these relations.

Figure 6 shows notched box plots illustrating the distribution of depression scores (BDI) across three alcohol consumption categories. Group 1 represents high consumption, Group 2 represents moderate consumption, and Group 3 represents occasional drinking. The median depression score was highest in the high-consumption group and lowest in the occasional-consumption group. The notches represent the 95% confidence interval for the medians; non-overlapping notches suggest statistically significant differences between groups. These results visually support the outcome of the ANOVA test, indicating group differences in depression severity based on alcohol use intensity.

This chart illustrates the ANOVA results upon comparing depression scores (BDI) depending on the alcohol consumption level (non-consumers, moderate consumers, and heavy consumers). The bars or the boxplots associated with each group show the median and dispersion of BDI scores, highlighting the significant differences statistically identified between categories.

**Figure 7:**
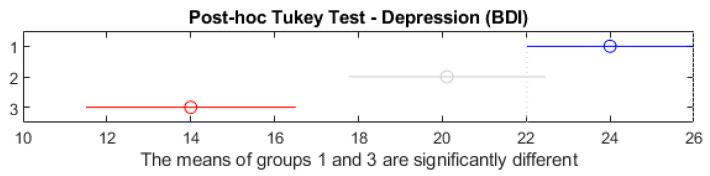
Post hoc Tukey test—depression.

Figure 7 presents the results of the post hoc BDI medium-comparison Tukey test between the three groups of alcohol consumers.

Figure 7 shows the post hoc Tukey HSD test comparing depression scores (BDI) across the three alcohol consumption groups. Red segments indicate statistically significant group differences (*p* < 0.05); blue segments denote non-significant comparisons (*p* ≥ 0.05). Horizontal lines represent 95% confidence intervals for the difference in group means. The test indicates a statistically significant difference between Group 1 (high consumption) and Group 3 (occasional consumption), as their confidence intervals do not overlap. No significant differences were observed between Groups 1 and 2 or Groups 2 and 3. This confirms that individuals with high alcohol consumption reported significantly higher depression levels than occasional drinkers.

In this chart, one can see the trust intervals of the medium differences and can precisely identify the group pairs in which significant differences occur (*p* < 0.05). The interpretation suggests that, in principle, the depression scores differ in non-consumers from those of high-alcohol-level consumers.

**Figure 8:**
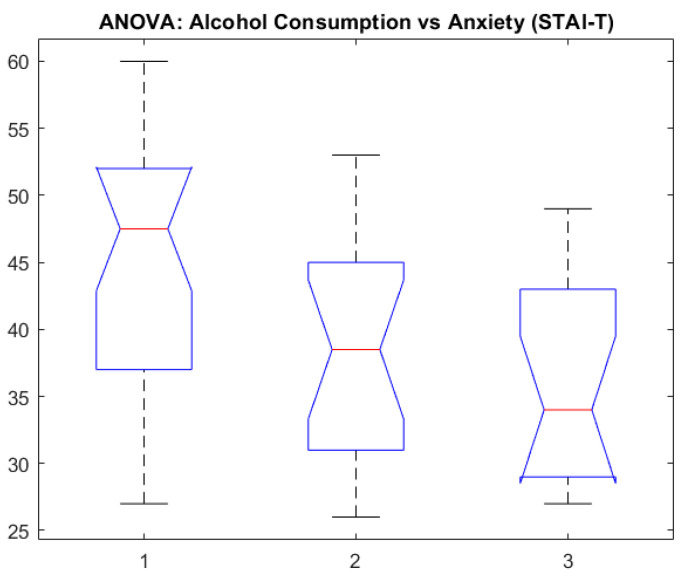
ANOVA: alcohol consumption vs. anxiety (STAI-T).

Figure 8 presents the association between alcohol consumption and trait anxiety (STAI_T).

Figure 8 exhibits notched boxplots showing the distribution of trait anxiety scores (STAI-T) across three groups based on alcohol consumption: Group 1—high; Group 2—moderate; Group 3—occasional. The median anxiety level is highest in the high-consumption group and lowest in the occasional group. The notches represent the 95% confidence intervals for the medians, suggesting statistically meaningful group differences where notches do not overlap. These results support a potential association between elevated alcohol use and higher trait anxiety levels.

## 4. Discussion

### 4.1. Interpreting the Main Findings

The results obtained after statistical analyses suggest the existence of a significant relationship between alcohol consumption and emotional changes in the young people included in the study. More precisely, we observed that increased alcohol consumption levels are associated with intensifying depressive (measured with BDI) and anxious symptoms (measured with STAI). This aspect supports the initial hypothesis according to which frequent exposure to alcohol can exacerbate emotional vulnerabilities, leading to enhanced depressive and anxious states.

First, ANOVA highlighted statistically significant differences between groups of young people with different alcohol consumption frequencies regarding depression scores. Although exact values can vary depending on the lot and the measurement instruments, the general trend indicates that the participants who consume alcohol regularly present a higher level of depressive symptoms compared to those who consume alcohol rarely or not at all. These results are in line with the specialty literature, which repeatedly documents that alcohol consumption, especially abusive or chronic consumption, can disturb emotional adjustment mechanisms and can increase the risk of emotional disorders.

Second, the positive correlation between alcohol consumption and anxiety confirms, at a practical level, that youngsters can appeal to alcohol as a dysfunctional coping strategy. Although alcohol is initially perceived as having an anxiolytic effect, longitudinal studies show that, overall, it can lead to an increased vulnerability to anxiety and the setting of a vicious circle—repeated consumption becomes both the cause and effect of emotional discomfort. Thus, the data obtained with the ANOVA method and with correlations indicate an increased risk of affecting the psychic balance of those developing a consistent consumption pattern.

Despite the observed group differences, the presence of negative correlations between alcohol use and emotional symptoms in the inferential analysis suggests possible underreporting, denial mechanisms, or alternative explanatory variables. These contradictory findings emphasize the need for caution in interpreting self-reported emotional states in individuals with chronic alcohol use and highlight the complexity of the relationships.

### 4.2. Discussing the Results Concerning the Initial Hypothesis

The research hypothesis started from the premise that youngsters with high alcohol consumption will face increased levels of depression and anxiety. The empirical results confirm this direction, suggesting that biological (for example, neurochemical vulnerability) and psychosocial factors (for example, group pressure, parental consumption models, and unresolved emotional trauma) can synergistically contribute to the onset and maintenance of consumption behavior. Also, the presence of emotional symptoms before consumption begins can accentuate the risk of using alcohol as a means of “self-medication” or escaping negative emotional states.

*The relationship between alcohol consumption and identified emotional changes and their practical significance*

From a practical perspective, the relationship between alcohol consumption and altered emotionality in young people has major implications in the field of prevention and psychological intervention. First, the results show the need for education and awareness programs specifically targeting adolescents and young adults, underlining the long-term risks of abusive alcohol consumption on mental health. Second, therapeutic interventions (such as cognitive-behavioral psychotherapy or emotional adjusting methods) should be integrated into wider strategies, which include periodic screening for depressive and anxious symptoms, together with an evaluation of the consumption degree.

Not least, the practical relevance of these findings manifests in the sphere of public health policies. Identifying the risk factors (for example, genetic vulnerability, lack of social support, and early exposure to alcohol) and protection factors (for example, social integration, mentoring programs, and family support) can provide guidance for the initiatives meant to reduce young people’s access to alcohol and offer them healthy alternatives for stress management. Creating campaigns that promote protective factors (resilience, coping abilities, and community support) could contribute to diminishing the incidence of emotional disorders associated with alcohol consumption.

The interpretation of the main findings suggests a potential relationship between alcohol consumption and emotional-state changes in young people. The seriousness of these findings calls for, on the one hand, multidisciplinary interventions that consider individual characteristics (e.g., genetic predispositions, psychosocial factors) and, on the other hand, a contextual approach in which family, school, and community actively contribute to maintaining young people’s psychological well-being.

### 4.3. Connections with Specialized Literature

This study’s results agree with previous evidence indicating a strong association between alcohol consumption and the increase in depressive and anxious symptoms in young people [21]. Numerous research studies [21,22,23,24] underline the fact that adolescents frequently exposed to alcohol can develop emotional adjustment vulnerabilities and an elevated risk of emotional disorders in the long run [25,26]. Our results are consistent with previous studies, which have evidenced that depression and anxiety can both precede and result from alcohol consumption. For instance, adolescents with higher levels of depressive and anxiety symptoms are at an increased risk of developing alcohol use disorders in young adulthood [27,28]. Conversely, alcohol use can exacerbate mental health issues, creating a vicious cycle. Studies have found that binge drinking may predict subsequent depressive symptoms, particularly in females. There are studies exposing that alcohol use can also dysregulate emotional processes, leading to heightened anxiety [29,30,31]. Anxiety sensitivity has been shown to mediate the relationship between depressive affects and substance use, suggesting that interventions targeting anxiety sensitivity can reduce both depression and alcohol use [32]. Another study conducted by Tiller (2013) highlighted that approximately 85% of patients with depression experience significant anxiety symptoms, and up to 90% of patients with anxiety disorders have comorbid depression. This comorbidity often leads to a more protracted illness course and less favorable treatment outcomes [33].

In particular, the neurobiological and environmental factors cooperate to amplify negative emotional responses [34], and the psychosocial stress characteristic of this moment in life can feed excessive consumption behavior [24,35,36,37,38,39,40]. Similar findings were observed in the Netherlands Study of Depression and Anxiety (NESDA), where 67% of individuals with depressive disorders had a comorbid anxiety disorder and 63% with anxiety disorders had comorbid depression. This study also noted that comorbidity was associated with factors like childhood trauma and higher neuroticism [41].

Longitudinal studies have identified both risk and protective factors associated with the co-occurrence of alcohol use and mental health issues. For example, delay of gratification has been found to differentiate between high and low co-occurrence groups, suggesting that interventions promoting self-regulation could mitigate risks [42]. Conversely, novelty-seeking behaviors have been linked to alcohol use trajectories, particularly in males [42,43].

The differences from other similar studies can be explained by a series of factors. First, sociocultural aspects play a key role, with proof that environments with more permissive norms toward alcohol consumption at an early age have a higher rate of binge drinking and a more pronounced association with emotional disorders [44]. Secondly, the sample’s characteristics (number of participants, gender distribution, or family history of psychiatric disorders) can determine the intensity and variety of documented emotional manifestations [45]. Also, the methodologies and evaluation instruments can lead to discrepancies in the results since self-declared data can be influenced by consumption underreporting or by the social acceptance level of alcohol among teenagers and young adults [46].

Despite these differences, the general convergence of our results with the specialized literature highlights the need to further investigate the relationship between alcohol consumption and emotionality, with particular attention to diverse cultural contexts and individual vulnerability factors. A more comprehensive understanding of these variables [47] will help develop more effective prevention and intervention strategies tailored to at-risk youth.

**Gender-Based Differences**

Although this study did not perform a separate statistical analysis by gender, previous research indicates that male and female adolescents may differ both in their alcohol consumption patterns and in the associated emotional outcomes. Females are more likely to use alcohol in response to internalizing symptoms, such as anxiety or depression, whereas males are more frequently associated with externalizing behaviors, including impulsivity and aggression, in the context of alcohol use [23,29,30].

Furthermore, adolescent girls who consume alcohol are at increased risk of developing persistent depressive symptoms over time, while alcohol use in boys is more commonly linked to conduct-related problems and sensation-seeking behavior [8,42].

These gender-specific patterns underscore the importance of incorporating sex as a moderating variable in future analyses and may help design tailored prevention strategies.

Although family and social contexts were not measured directly, their potential influence was considered important and was discussed based on existing findings.

Integrated treatment approaches that address both mental health and substance use disorders have shown promise. Cognitive behavioral therapy (CBT) and motivational interviewing are effective in reducing both anxiety and alcohol use [48,49]. Digital interventions, such as the DEAL Project, have demonstrated short-term efficacy in reducing both depressive symptoms and alcohol use, though long-term sustainability remains a challenge [50].

**Applicability of the study**

Understanding the correlations between alcohol use, depression, and anxiety among young adults is crucial due to the significant public health implications. This demographic is particularly vulnerable to mental health disorders and substance use, which can adversely affect their emotional development, academic performance, interpersonal relationships, and long-term functioning.

The findings of this study may contribute to the early identification of at-risk individuals and guide the design of tailored psychological interventions. Furthermore, they can inform public health policies aimed at promoting emotional regulation and preventing harmful alcohol use among adolescents and young adults. Integrating these insights into educational and clinical settings may improve screening procedures, facilitate timely referrals, and enhance treatment engagement.

**Limitations of the Study**

This study has several limitations that should be acknowledged. First, the small sample size (N = 60) may limit the statistical power and generalizability of the findings. The reduced number of participants also restricts the capacity to conduct subgroup analyses or detect more subtle effects and interactions between variables. As this was a pilot study, no power analysis was conducted beforehand; this will be addressed in a future, larger-scale investigation.

Second, the cross-sectional nature of the study does not allow for causal inferences regarding the directionality of the relationships between alcohol consumption, depression, and anxiety. Longitudinal studies are needed to determine how these variables evolve and influence one another.

Third, the data were primarily based on self-reported questionnaires, which may be affected by social desirability bias or inaccurate recall, particularly regarding alcohol consumption. Although biological markers were used to complement these assessments, their interpretation is limited without additional clinical or biochemical data.

Another limitation relates to the study period (2021–2023), which overlapped with the COVID-19 pandemic and associated lockdowns. These external stressors may have influenced participants’ emotional state independently of alcohol use. While we could not isolate these effects statistically, their potential impact is acknowledged and should be addressed in future controlled studies.

Finally, potential confounding variables, such as a family history of psychiatric disorders, social support, coping mechanisms, or concurrent medication use, were not controlled for, which may have influenced the results.

**Future research directions**

The first necessary step to examine the relationship between alcohol consumption and emotional changes in young people is to initiate longitudinal studies, which allow us to trace emotional symptoms during the transition period toward maturity. This approach would offer more precise information about the temporal sequence of emotional changes and consumption behavior, thus helping to identify risk factors and resilience mechanisms that can appear during various stages of maturation. Another aspect worth investigating targets a more detailed analysis of specific variables, like gender and the socio-economic situation; the literature suggests that children can present different response patterns to alcohol consumption, and the socioeconomic environment can moderate access to supporting resources or amplify psychosocial stress. Studying these subgroups could show key features in emotional manifestations and consumption models.

Given the cultural context diversity and their role in normalizing or discouraging alcohol consumption at an early age, we must start some sort of intercultural research. Comparing results from different countries and regions would contribute to a more nuanced understanding of the way social norms and community values influence the start and evolution of such problematic consumption. Moreover, using some experimental designs in which we can control variables such as stress factor exposure or offering specific psychological interventions could clarify the causative mechanisms through which alcohol influences emotional states. Such an approach would allow us to evaluate the efficiency of different prevention and treatment methods, contributing to the development of intervention programs more focused on the real needs of the young population and the establishment of scientifically based public policy strategies.

## 5. Conclusions

This study’s results suggest that the frequency and quantity of alcohol consumption in young people may be associated with increased emotional vulnerability, particularly regarding depressive and anxious symptoms. These associations appear to be influenced by both neurobiological vulnerabilities specific to this developmental stage and by social and emotional pressures.

The practical relevance of these findings consists of highlighting the need for health policies and specific programs to prevent and fight excessive alcohol consumption in adolescents and young adults. By including aspects concerning mental health in educational programs and providing easy access to psychological counseling, we can reduce the risk of emotional disorders associated with the use of alcohol and its chronic consumption.

This study underlines the importance of early prevention and intervention programs. Multidisciplinary support, which can integrate both the psychoeducational component and medical and psychotherapeutic interventions, is crucial in helping young people identify and manage the risk factors more efficiently, diminishing the negative consequences of alcohol consumption on their emotional health in the long term.

This study’s results suggest that alcohol consumption may be associated with elevated depressive and anxious symptoms in young people. However, due to contradictory statistical findings and the study’s exploratory nature, these associations should be interpreted with caution and further validated in larger, longitudinal samples.

## Figures and Tables

**Table 1:** Summary of descriptive statistics for age, depression, and anxiety scores.

VARIABLE	MEAN (M)	STANDARD DEVIATION (SD)	95% CONFIDENCE INTERVAL
AGE (YEARS)	24.18	3.80	-
BDI (DEPRESSION)	15.72	6.89	[13.09, 17.50]
STAI_S (ANXIETY)	40.27	8.59	[37.95, 42.58]

## Data Availability

This paper is part of the doctoral study of Psychologist Simona-Dana Mitincu-Caramfil: “The role of ethanolic consumption in depressive syndrome development”.

## Abbreviations

The following abbreviations are used in this manuscript:

FFM	Five-Factor Model;
BDI	Beck Depression Inventory;
STAI	State-Trait Anxiety Inventory;
STAI_S	State Anxiety Inventory;
STAI_T	Trait Anxiety Inventory;
AffectPoz	Scores for the positive emotionality;
AffectNeg	Scores for the negative emotionality.
